# Stochastic Uncertainty Analysis of Integrated Blisk–Shaft Rotor Vibrations Using Artificial Neural Networks and Reduced-Order Models

**DOI:** 10.3390/ma19040696

**Published:** 2026-02-12

**Authors:** Hongyun Sun, Xinqi Li, Xinjie Bai, Huiqun Yuan, Hongyuan Zhang

**Affiliations:** 1 School of Automotive and Transportation, Shenyang Ligong University, Shenyang 110159, China; 15715380361@163.com (X.L.); 17832875307@163.com (X.B.); 2 College of Sciences, Northeastern University, Shenyang 110819, China; yuan_hq@163.com

**Keywords:** integrated blisk–shaft rotor, stochastic uncertainty analysis, reduced-order modeling, artificial neural network, modal sensitivity analysis

## Abstract

Integrated blisk–shaft rotors represent a critical advancement in aero-engine design, offering enhanced structural integrity and weight reduction. However, their complex dynamic behavior under inherent material uncertainties poses significant challenges for reliable vibration prediction. This study presents a novel stochastic uncertainty analysis framework combining reduced-order finite element modeling and artificial neural networks (ANNs) to efficiently and accurately quantify the modal variability of integrated blisk–shaft rotors. A high-fidelity finite element model is first developed, followed by the construction and validation of a reduced-order model (ROM) to substantially decrease computational costs while preserving modal accuracy. Material parameter uncertainties are introduced, and corresponding natural frequencies are computed using the ROM. Subsequently, an ANN surrogate model is trained to capture the nonlinear mapping between uncertain input parameters and modal frequencies, enabling rapid prediction across the stochastic parameter space. The proposed approach is employed to perform comprehensive uncertainty propagation and global sensitivity analyses, identifying the dominant parameters influencing each modal frequency. Results demonstrate that the combined ROM-ANN methodology achieves high predictive accuracy with significantly reduced computational effort, offering an effective tool for uncertainty-aware dynamic analysis and design optimization of integrated blisk–shaft rotors. This work advances the integration of machine learning techniques with classical structural dynamics for robust aero-engine rotor design under uncertainty.

## 1. Introduction

In modern aero-engine design, integrated blisk–shaft rotors have attracted significant attention due to their superior structural integrity, reduced weight, and improved dynamic performance compared to traditional assembled rotor systems [1,2]. By combining the compressor or turbine disk (blisk) and the shaft into a monolithic component, this novel configuration effectively eliminates interface-induced issues such as joint looseness and stress concentrations, which are common in conventional disk–shaft assemblies [3,4,5]. Consequently, integrated blisk–shaft rotors offer enhanced reliability, longer fatigue life, and simplified maintenance, making them a key development direction for next-generation aero-engines. However, the dynamic behavior of these integrated structures is inherently complex due to strong coupling effects between the disk and shaft, and this complexity is further exacerbated by the presence of uncertainties in material properties, geometric parameters, and manufacturing processes [6,7]. Moreover, operating conditions may introduce stress-stiffening (prestress) effects that can alter modal characteristics and coupling, thereby further increasing design difficulty [8].

Accurate prediction of modal characteristics such as natural frequencies and mode shapes is essential for understanding vibration behavior and ensuring structural integrity. Traditional finite element (FE) methods have been widely employed to capture the detailed modal characteristics of blisks and rotor assemblies under deterministic conditions [9,10,11,12]. Early studies demonstrated the effectiveness of high-fidelity FE modeling and component mode synthesis (CMS) techniques in reducing computational cost while maintaining acceptable accuracy. However, classical deterministic analyses neglect the unavoidable uncertainties present in actual engineering applications.

In recent years, increasing research attention has been devoted to quantifying the influence of uncertainties—including manufacturing tolerances, material variability, and wear—on the dynamic behavior of turbomachinery structures. Stochastic finite element methods (SFEM), perturbation techniques, and Monte Carlo simulations have been widely adopted to analyze how random variations in stiffness, density, and damping affect blisks and rotor systems [13,14,15]. These studies have shown that uncertainties can cause significant variations in modal properties, leading to unpredictable vibration responses and potential structural failures. Nevertheless, for turbomachinery rotors, most existing studies are limited to separate blade-disk or blisk assemblies; the stochastic modal behavior of fully integrated blisk–shaft rotors remains largely unexplored, despite their distinct structural coupling mechanisms and uncertainty propagation pathways [16,17,18]. This gap motivates dedicated research on integrated structures and is further corroborated by a recent state-of-the-art review on rotor-system uncertainty analysis, which emphasizes that integrated multi-component coupling and operating-condition effects remain challenging topics for practical UQ [19].

From a computational perspective, incorporating uncertainties into modal analysis requires repeated simulations—often thousands of FE evaluations in a Monte Carlo framework. High-fidelity FE models of integrated blisk–shaft rotors typically contain hundreds of thousands to millions of degrees of freedom, making direct stochastic simulation computationally infeasible [20,21,22]. Reduced-order modeling (ROM) techniques such as Craig–Bampton CMS, modal truncation, and component mode synthesis have been proposed to alleviate this issue. ROM has demonstrated impressive performance in deterministic rotor analysis and even in some uncertainty analyses for blisks [23,24,25,26]. However, the accuracy and robustness of ROM under wide-range stochastic perturbations for strongly coupled integrated blisk–shaft rotors have not been comprehensively validated. This concern is also echoed in recent rotor dynamics research, which points out that ROM robustness under random perturbations is nontrivial and should be examined when ROM is used for UQ [27].

To further improve computational efficiency, surrogate modeling techniques have been widely studied. Polynomial chaos expansion (PCE), Kriging, and radial basis functions (RBF) have been applied to approximate the mapping between uncertain parameters and modal outputs [28,29,30,31]. However, these classical surrogate models often face limitations when dealing with high-dimensional, nonlinear stochastic mappings inherent in integrated rotor dynamics. Artificial neural networks (ANNs), known for their excellent nonlinear approximation ability, have recently gained popularity as surrogate models in vibration prediction, structural uncertainty quantification, fault diagnosis and fatigue evaluation [32,33,34]. ANN-based surrogates have been successfully applied to mistuned blisks and damaged rotor systems, demonstrating strong potential for dynamic prediction under uncertainty.

Beyond uncertainty quantification, understanding the relative influence of uncertain parameters is critical for optimizing design and manufacturing processes. Global sensitivity analysis, including Sobol indices and ANN-assisted techniques, has been applied to wind turbine structures and rotor balancing problems [35,36,37]. However, few studies have incorporated sensitivity analysis specifically for integrated blisk–shaft rotors within a computationally efficient surrogate framework.

To address these gaps, this paper proposes a comprehensive stochastic uncertainty analysis framework for integrated blisk–shaft rotors by combining reduced-order FE modeling with ANN surrogate modeling. In contrast to many existing studies that focus on isolated blisks or simplified rotor models, this work targets an integrated blisk–shaft configuration with strong disk–blade–shaft coupling under operating conditions. First, a high-fidelity FE model is constructed to capture the detailed dynamic behavior of the integrated rotor. A reduced-order model is then developed and rigorously validated against the full-order model to ensure accuracy under material uncertainties. Probabilistic variations in elastic modulus, density, and Poisson’s ratio are introduced to generate random samples, and the ROM is used to efficiently compute natural frequencies. An ANN surrogate model is trained to learn the nonlinear parameter–frequency mapping, enabling rapid prediction across the stochastic parameter space. Subsequently, uncertainty propagation and global sensitivity analyses are performed to quantify modal variability and identify dominant parameters, providing valuable insights for robust rotor design and tolerance optimization. The key methodological contributions are: (i) a prestress-consistent reduced-order modeling strategy tailored to integrated blisk–shaft coupling; (ii) a ROM-driven ANN surrogate workflow enabling efficient high-dimensional uncertainty propagation; and (iii) a unified UQ–sensitivity study that quantifies uncertainty transmission and parameter importance for the most critical coupled modes.

The remainder of this paper is organized as follows: Section 2 details the finite element modeling and reduced-order modeling approach for the integrated blisk–shaft rotor. Section 3 describes the uncertainty modeling, stochastic sampling strategies, and construction of the ANN surrogate. Section 4 presents the uncertainty propagation results and sensitivity analyses. Finally, Section 5 summarizes the key findings and discusses future research directions.

## 2. Finite Element Modeling and Reduced-Order Formulation of the Integrated Blisk–Shaft Rotor

### 2.1. Structural Configuration of the Integrated Blisk–Shaft Rotor

**Figure 1:**
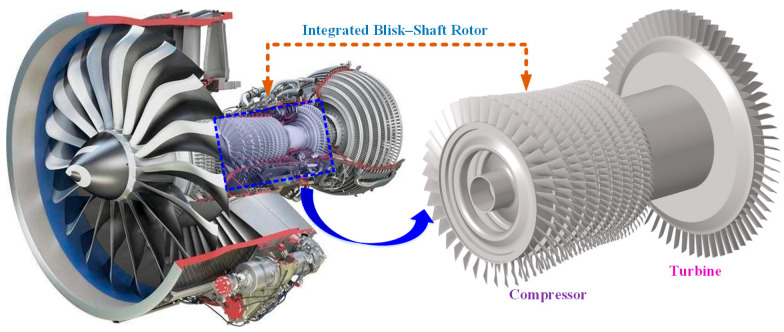
Structural configuration of the integrated blisk–shaft rotor in an aeroengine.

The integrated blisk–shaft rotor investigated in this study represents a typical configuration employed in modern aero-engine compressors and turbines, in which the bladed disk (blisk) and the shaft are manufactured as a single monolithic structure. As illustrated in Figure 1, the rotor consists of multiple blisk stages rigidly connected to a central shaft, forming an integrated rotating assembly spanning the compressor and turbine sections. Compared with conventional assembled disk–shaft rotors, this integrated configuration eliminates mechanical joints such as bolted or interference-fit interfaces, thereby significantly improving structural integrity and ensuring continuous load transfer along the rotor axis.

Each blisk stage comprises a disk body and circumferentially distributed blades that are integrally machined with the disk. The shaft serves as the primary torque-transmission component and provides axial support for the entire rotating assembly. Owing to the monolithic design, the blisk stages and the shaft exhibit continuous material properties and geometric compatibility, resulting in strong structural coupling among bending, torsional, and axial deformation modes. This coupling characteristic is a defining feature of integrated blisk–shaft rotors and has a pronounced influence on their dynamic behavior.

In the present configuration, the rotor includes ten blisk stages with non-uniform blade counts along the axial direction, reflecting typical design practices in aero-engine compressors and turbines. The number of blades in each stage is summarized in Table 1. Specifically, the blade count increases progressively from the upstream stages to the mid-stages and then decreases toward the downstream stages, leading to variations in structural periodicity and stiffness distribution across the rotor. Such non-uniformity in blade numbers introduces additional complexity to the modal characteristics and further enhances inter-stage dynamic coupling.

### 2.2. Finite Element Modeling of the Integrated Blisk–Shaft Rotor

The integrated blisk–shaft rotor is a geometrically complex, multi-stage rotating structure in which each blisk stage is characterized by a distinct blade count and structural periodicity. Owing to this non-uniform circumferential configuration, a global finite element model cannot be constructed from a single fundamental sector without violating the assumptions of cyclic symmetry. Conversely, direct full-geometry meshing of the entire rotor leads to severe mesh non-uniformity and numerical inconsistencies, which may compromise the accuracy of vibration predictions.

**Figure 2:**
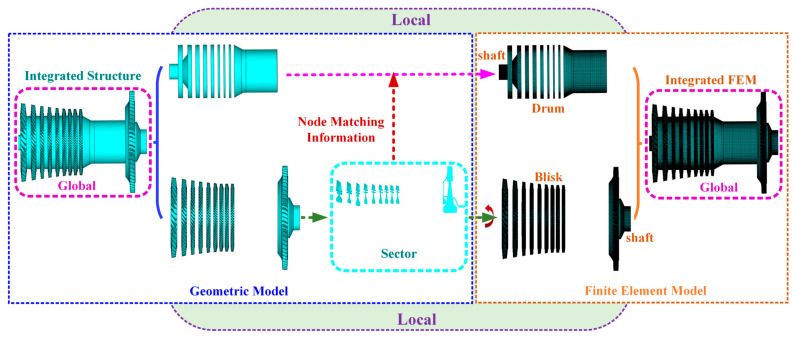
Schematic of the global–local–global finite element modeling procedure for the integrated blisk–shaft rotor.

To overcome these challenges, a global–local–global finite element modeling strategy is adopted, as schematically illustrated in Figure 2. In this approach, the integrated rotor is first decomposed into blisk stages, drums, and shaft segments. Each blisk stage is independently discretized using a representative sector under the assumption of ideal cyclic symmetry, and the full blisk geometry is reconstructed through circumferential replication. This local modeling process ensures consistent mesh quality within each stage while preserving geometric fidelity.

Because adjacent stages differ in sector numbers, the node distributions at the interfaces between blisks, drums, and shaft segments are inherently nonconforming. To ensure structural continuity, transition elements are introduced to achieve node compatibility while maintaining smooth mesh grading across interfaces. The fully discretized substructures are then assembled into a unified finite element model, resulting in a dynamically consistent representation of the integrated blisk–shaft rotor suitable for high-fidelity modal and stochastic analyses.

The material properties of the compressor blades, compressor disks, and turbine blades and disks are explicitly defined to ensure physical realism. The corresponding material parameters employed in this study are summarized in Table 2.

### 2.3. Reduced-Order Modeling (ROM)

#### 2.3.1. Multi-Level Substructuring Strategy

Due to the large scale and strong inter-stage coupling characteristics of the integrated blisk–shaft rotor, a direct reduced-order modeling of the full finite element model is generally ineffective. On the one hand, the extremely high number of degrees of freedom results in prohibitive computational cost; on the other hand, conventional single-level reduction strategies often fail to preserve the complex dynamic interactions among blades, disks, drums, and the shaft. In particular, the coexistence of multiple blisk stages with different blade counts and strong structural coupling makes it difficult to achieve a satisfactory balance between computational efficiency and dynamic fidelity using traditional component mode synthesis approaches.

**Figure 3:**
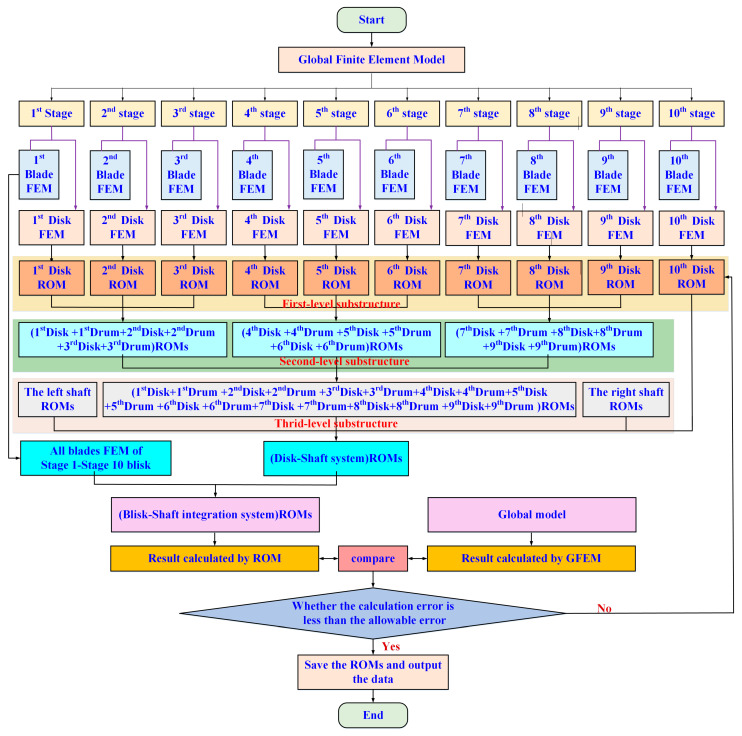
The multi-stage prestressed substructuring reduced-order modeling framework for integrated blisk–shaft rotors.

To address these challenges, a multi-level substructuring strategy is adopted in this study. The integrated blisk–shaft rotor is decomposed into a hierarchy of physically meaningful substructures, enabling staged model reduction while preserving essential dynamic characteristics across different structural levels. The overall framework of the proposed multi-level prestressed substructuring reduced-order modeling approach is illustrated in Figure 3, which schematically presents the decomposition, reduction, and assembly procedures from local components to the global reduced-order model.

At the first reduction level, the integrated rotor is partitioned into single-stage blisk substructures, where each stage consists of circumferentially distributed blades and the corresponding disk. The drum segments connecting adjacent stages are treated as interface boundaries. At this level, the blade degrees of freedom are fully retained, as blade dynamics dominate the vibration response of the rotor and play a critical role in modal characteristics. This decomposition allows the local dynamic behavior of each blisk stage to be represented independently while ensuring compatibility at the inter-stage interfaces.

At the second reduction level, neighboring disk–drum substructures are further grouped into multi-stage disk–drum assemblies. This hierarchical aggregation explicitly accounts for inter-stage coupling effects introduced by the continuous shaft and drum connections, which cannot be captured by single-stage models alone. By condensing internal degrees of freedom within these assemblies, the overall system size is significantly reduced while maintaining the continuity of displacement and force transmission across interfaces.

At the third reduction level, the left and right shaft segments are incorporated as separate substructures and subsequently coupled with the reduced disk–drum assemblies. This final assembly step yields a global reduced-order model of the integrated blisk–shaft rotor that captures the coupled dynamic behavior of the entire system. Through this staged reduction process, the original large-scale finite element model is systematically transformed into a compact reduced-order representation with controlled accuracy.

As shown in Figure 3, the proposed multi-level substructuring strategy provides a clear physical interpretation of the reduction process and establishes a structured pathway from local component dynamics to global system behavior. More importantly, this framework creates a natural foundation for the consistent incorporation of prestress effects and modal information transfer across different structural levels, which is essential for accurate dynamic prediction of integrated blisk–shaft rotors operating under high rotational speeds.

While the multi-level substructuring strategy described in Section 2.3.1 provides a clear physical decomposition of the integrated blisk–shaft rotor, an accurate reduced-order representation further requires a consistent treatment of prestress effects and modal information across different structural levels. Under operating rotational speeds, centrifugal forces introduce significant prestress states in blades, disks, and shaft segments, which modify the effective stiffness distribution and alter the modal characteristics of each substructure. If such prestress effects are neglected or inconsistently treated during model reduction, substantial errors may arise in both natural frequencies and mode shapes, particularly for low-order global modes that dominate the coupled vibration behavior. Therefore, a prestress-consistent modal extraction and transfer procedure is essential to ensure that the reduced-order model faithfully preserves the dynamic characteristics of the integrated rotor. This motivates the prestress-consistent modal extraction and transfer method presented in the following section.

#### 2.3.2. Prestress-Consistent Modal Extraction and Transfer Method

(1) Prestressed substructural governing equations

For the *i*-th substructure of the integrated blisk–shaft rotor, the linearized equation of motion under rotational prestress can be written as

(1) $$M_{i}{\overset{‥}{u}}_{i}+\left(K_{i}+K_{i}^{\mathrm{cf}}-K_{i}^{\mathrm{rs}}\right)u_{i}=f_{i}$$

where $M_{i}$ is the mass matrix, $K_{i}$ is the elastic stiffness matrix, $K_{i}^{\mathrm{cf}}$ and $K_{i}^{\mathrm{rs}}$ denote the centrifugal stiffening and rotational softening matrices, respectively, $u_{i}$ is the physical displacement vector.

For brevity, a prestress-augmented stiffness matrix is introduced:

(2) $${\tilde{K}}_{i}=K_{i}+K_{i}^{\mathrm{cf}}-K_{i}^{\mathrm{rs}}$$

(2) Interface–interior partitioning

The displacement vector is partitioned into interior and interface degrees of freedom (DOFs):

(3) $$u_{i}=\left[\begin{array}{c} u_{i}^{I}\\ u_{i}^{B} \end{array}\right],\;{\tilde{K}}_{i}=\left[\begin{array}{cc} {\tilde{K}}_{ii} & {\tilde{K}}_{ib}\\ {\tilde{K}}_{bi} & {\tilde{K}}_{bb} \end{array}\right]$$

Under fixed-interface conditions ($u_{i}^{B}=0$), the prestressed fixed-interface eigenproblem is formulated as

(4) $$\;\left({\tilde{K}}_{ii}-\omega^{2}M_{ii}\right)\Phi_{i}^{\mathrm{FI}}=0$$

Solving Equation (4) yields the complete modal basis

(5) $$\Phi_{i}^{\mathrm{FI}}=\left[\begin{array}{ll} \Phi_{i}^{L} & \Phi_{i}^{H} \end{array}\right],$$

where $\Phi_{i}^{L}$ and $\Phi_{i}^{H}$ denote the retained low-order and truncated high-order prestressed modes, respectively.

(3) Prestress-consistent Ritz transformation

Retaining only the dominant low-order prestressed modes, the interior displacement is approximated as

(6) $$u_{i}^{I}\approx \Phi_{i}^{L}q_{i}$$

leading to the following prestress-consistent Ritz transformation:

(7) $$u_{i}=\left[\begin{array}{cc} \Phi_{i}^{L} & 0\\ 0 & I \end{array}\right]\left[\begin{array}{c} q_{i}\\ u_{i}^{B} \end{array}\right]=T_{i}z_{i}$$

Substituting Equation (7) into Equation (1) and applying Galerkin projection yields the reduced prestressed substructural equations

(8) $$M_{i}^{r}{\ddot{z}}_{i}+K_{i}^{r}z_{i}=f_{i}^{r}$$

With

(9) $$M_{i}^{r}=T_{i}^{T}M_{i}T_{i},\;K_{i}^{r}=T_{i}^{T}K_{i}T_{i}$$

(4) Prestress-consistent modal transfer and assembly

For two adjacent substructures *i* and *j*, compatibility and equilibrium at the shared interface require

(10) $$u_{i}^{B}=u_{j}^{B},\;f_{i}^{B}+f_{j}^{B}=0$$

By enforcing Equation (10) through constraint transformation or Boolean assembly matrices, all reduced substructures are assembled into a global prestressed reduced-order model:

(11) $$M^{\mathrm{ROM}}\ddot{p}+K^{\mathrm{ROM}}p=0$$

where the reduced coordinates ***p*** represent the coupled modal participation of blades, disks, drums, and shaft

Solving Equation (11) yields the global prestress-consistent modal characteristics of the integrated blisk–shaft rotor, which are subsequently validated through frequency error and MAC evaluations in Section 2.3.3.

#### 2.3.3. ROM Error Evaluation and Validation Metrics

Following the establishment of the multi-level substructuring strategy in Section 2.3.1 and Section 2.3.2, it is essential to quantitatively assess the accuracy and reliability of the resulting reduced-order model (ROM). In this study, the fidelity of the proposed prestressed multi-level ROM is systematically evaluated by comparing its modal characteristics with those obtained from the corresponding global finite element model (FEM). Two complementary validation metrics are employed: natural frequency error and the Modal Assurance Criterion (MAC).

The natural frequency error provides a direct measure of the deviation between the ROM-predicted eigenfrequencies and the reference solutions obtained from the GFEM under identical prestress conditions. For the *k*-th mode, the relative frequency error is defined as

(12) $$\varepsilon_{k}^{\left(f\right)}=\frac{\left|f_{k}^{\mathrm{ROM}}-f_{k}^{\mathrm{FEM}}\right|}{f_{k}^{\mathrm{FEM}}}\times 100\%$$

where $f_{k}^{\mathrm{ROM}}$ and $f_{k}^{\mathrm{FEM}}$ denote the *k*-th natural frequency computed from the reduced-order model and the global finite element model, respectively.

This metric directly reflects the capability of the ROM to preserve the global stiffness–mass characteristics of the integrated blisk–shaft rotor, including the effects of centrifugal stiffening and rotational softening introduced in Section 2.3.2. In practical applications, acceptable accuracy is typically achieved when the relative frequency error of the dominant low-order modes remains within a few percent.

**Figure 4:**
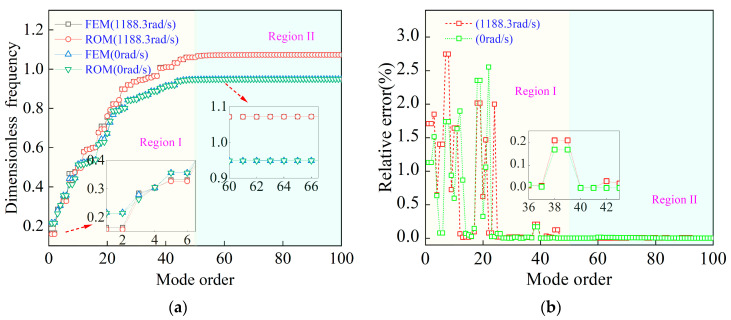
Validation of the reduced-order model: (**a**) comparison of dimensionless natural frequencies and (**b**) relative frequency errors between FEM and ROM under different rotational speeds.

Following the formulation of the multi-level substructuring strategy and the prestress-consistent modal transmission described in Section 2.3.1 and Section 2.3.2, the accuracy of the proposed reduced-order model is quantitatively assessed against the global finite element model (FEM). Figure 4 compares the dimensionless natural frequencies and corresponding relative frequency errors under both zero-speed and operating-speed (1188.3 rad/s) conditions.

As shown in Figure 4a, the modal spectrum can be clearly divided into two regions. Region I (approximately modes 1–50) is dominated by global bending and coupled disk–shaft deformation. In this region, the ROM predictions closely match the GFEM results at both rotational speeds, with relative frequency errors remaining below approximately 2–3%, as shown in Figure 4b. This confirms that the proposed prestressed multi-level reduction accurately preserves the global stiffness–mass characteristics of the integrated rotor, including centrifugal stiffening effects.

Region II (modes above approximately 50) corresponds primarily to higher-order local blade-dominated modes. In this region, the relative frequency errors rapidly decrease to near zero, indicating that the retained blade degrees of freedom and hierarchical substructuring effectively capture local dynamic behavior without introducing numerical artifacts.

Overall, the proposed prestressed multi-level ROM demonstrates high fidelity across the full modal range of interest, with particularly strong accuracy in the low-order modes most relevant to vibration analysis and uncertainty quantification. This validation provides a reliable foundation for the stochastic analyses presented in Section 3.

While frequency agreement is necessary, it is not sufficient to fully characterize the accuracy of a reduced-order model, particularly for strongly coupled multi-stage rotor systems. Therefore, the similarity of mode shapes between the ROM and FEM is further quantified using the Modal Assurance Criterion (MAC), defined as

(13) $${\mathrm{MAC}}_{k}=\frac{{\left(\phi_{k}^{\mathrm{ROMT}}\phi_{k}^{\mathrm{FEM}}\right)}^{2}}{\left(\phi_{k}^{\mathrm{ROMT}}\phi_{k}^{\mathrm{ROM}}\right)\left(\phi_{k}^{\mathrm{FEMT}}\phi_{k}^{\mathrm{FEM}}\right)}$$

where $\phi_{k}^{\mathrm{ROM}}$ and $\phi_{k}^{\mathrm{FEM}}$ represent the *k*-th normalized blade mode shape vectors obtained from the reduced-order and global finite element models, respectively.

The MAC value ranges from 0 to 1, with values close to unity indicating strong correlation between mode shapes. In the context of integrated blisk–shaft rotors, a high MAC value confirms that the proposed multi-level prestressed substructuring strategy successfully preserves the coupled bending–torsional–axial deformation patterns across blades, disks, drums, and shafts.

**Figure 5:**
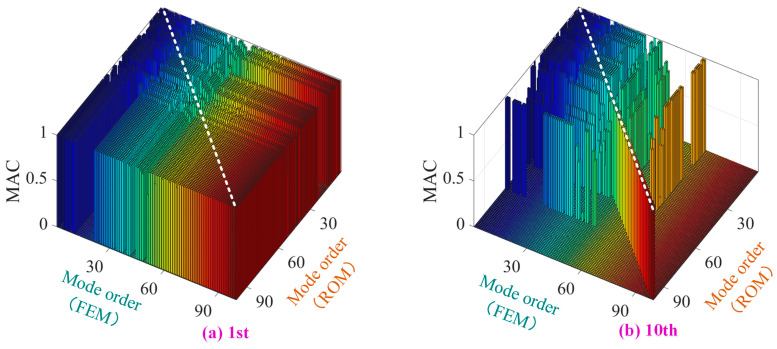
Modal Assurance Criterion (MAC) matrices between FEM and ROM for the 1st and 10th blisk stages.

Figure 5 presents representative MAC matrices for the first-stage and tenth-stage blisk substructures. For the first-stage blisk, the MAC matrix exhibits a pronounced diagonal dominance, with the majority of diagonal values exceeding 0.9, indicating that the proposed prestressed multi-level ROM accurately preserves the global bending and disk–shaft coupled modes that dominate the low-order dynamics. Minor reductions in MAC values for a few higher-order modes are primarily associated with local blade-dominated modes, where slight discrepancies in modal localization may occur.

Similarly, the MAC results for the tenth-stage blisk demonstrate excellent modal correlation, with diagonal MAC values consistently above 0.9 across the retained modal range. This confirms that the hierarchical substructuring strategy and prestress-consistent modal transmission effectively capture the dynamic characteristics of downstream stages, despite differences in blade count, structural periodicity, and local stiffness distribution.

For the intermediate stages (Stages 2–9), although individual MAC matrices are not explicitly shown for brevity, all principal diagonal MAC values are verified to be greater than 0.9. This uniformly high level of modal correlation across all stages confirms the robustness and consistency of the proposed reduced-order modeling framework throughout the entire integrated blisk–shaft rotor.

From a computational efficiency perspective, the proposed reduced-order modeling strategy leads to a significant reduction in system size and computational cost. The global finite element model (FEM) of the integrated blisk–shaft rotor contains 2,594,595 degrees of freedom, whereas the reduced-order model (ROM) retains 632,149 degrees of freedom after substructuring and modal truncation, corresponding to a DOF reduction of approximately 75.6%.

Under the same computational environment (11th Gen Intel^®^ Core™ i7-11700, 32 GB RAM, Windows 10 64-bit), computing the first 100 natural modes using the FEM requires approximately 24.5 h, while the corresponding ROM-based analysis is completed in about 4.2 h. This represents a computational speed-up of nearly 5.83 times for high-order modal extraction. Such efficiency gains are critical for enabling repeated stochastic simulations and surrogate-model training for integrated blisk–shaft rotors, which would otherwise be computationally prohibitive using the full-order model.

To further demonstrate robustness for stochastic applications, additional validation cases are performed under an intermediate rotational speed (Ω = 500 rad/s) and representative perturbations of key material parameters within the uncertainty range considered (±2% in the Young’s modulus of the turbine disk and density of the compressor blade). Table 3 summarizes the maximum relative frequency errors and the minimum MAC values for the dynamically critical low-order modes. The consistently low frequency errors and high MAC values confirm that the proposed prestress-consistent multi-level ROM remains accurate and stable across the extended operating and parameter conditions, supporting its use for uncertainty propagation.

Overall, the combined frequency error and MAC evaluations demonstrate that the prestressed multi-level ROM accurately predicts both natural frequencies and mode shapes. The extended validation cases in Table 3 further confirm that this accuracy is maintained under an additional rotational speed and representative material perturbations, supporting robustness for stochastic applications. This provides a reliable foundation for the subsequent uncertainty and sensitivity analyses.

### 2.4. Modal Characteristics and Coupled Vibration Mechanisms of the Integrated Blisk–Shaft Rotor

Following the quantitative validation of the proposed reduced-order model in Section 2.3 through natural frequency errors and MAC indices, the present section focuses on the physical interpretation of the vibration behavior of the integrated blisk–shaft rotor. Using the global finite element model as a high-fidelity reference, the modal characteristics are systematically analyzed to reveal the coupled vibration mechanisms, repeated modes, and prestress-induced effects that govern the dynamic response of the integrated system.

#### 2.4.1. Global Modal Classification and Coupled Mode Shapes

The global mode shapes of the integrated blisk–shaft rotor exhibit pronounced multi-component coupling characteristics that fundamentally distinguish them from conventional single-blisk or isolated disk–shaft systems.

**Figure 6:**
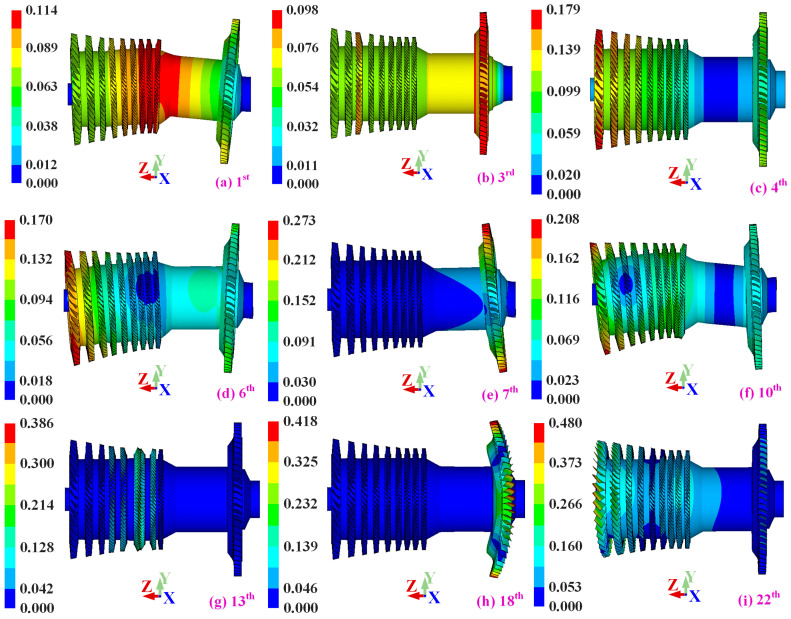
Mode Shape of integrated blisk–shaft rotors system.

As illustrated in Figure 6 and summarized in Table 4, the low-order modes are dominated by global shaft–disk coupled bending and axial translational motion, in which blades, disks, and shaft deform coherently as an integrated structure. These modes are characterized by long axial wavelengths and strong inter-stage correlation, indicating that the vibration energy is governed by the global stiffness and mass distribution of the rotor.

With increasing modal order, the vibration patterns evolve into more complex coupled forms, including global torsional modes, bending–torsion coupled modes, and blade-dominated local deformation superimposed on global motion. Notably, vibration forms such as global axial motion and bending–torsion coupling—often neglected in single-blisk analyses—become clearly identifiable in the integrated configuration, demonstrating that single-stage or component-level models are insufficient to capture the full modal topology of aero-engine rotors.

A salient feature of the integrated blisk–shaft rotor is the presence of closely spaced and repeated (near-degenerate) natural frequencies, as evidenced in both zero-speed and operating-speed conditions.

These repeated modes originate from the combined effects of circumferential symmetry at the blisk level and axial continuity of the shaft, allowing multiple global vibration patterns to share nearly identical eigenfrequencies. Under operating rotational speeds, centrifugal prestress further modifies the effective stiffness of blades and disks, leading to frequency splitting, mode veering, and redistribution of modal energy between shaft-dominated and blade-dominated components.

Importantly, prestress does not merely shift modal frequencies but fundamentally alters the modal coupling mechanisms across structural scales. As a result, vibration behaviors such as global torsional motion and bending–torsion interaction become dynamically significant, even though they may be weak or absent in unstressed or single-blisk models. This observation highlights the necessity of a prestress-consistent, fully integrated modeling framework for accurately resolving repeated modes and predicting the true vibration characteristics of aero-engine rotors under realistic operating conditions.

#### 2.4.2. Stage-Resolved Coupling Analysis Based on BCNDS

To further quantify the coupled vibration topology of the integrated blisk–shaft rotor beyond conventional mode-shape inspection, we introduce the blade characteristic nodal-diameter spectrum (BCNDS) concept [38], originally developed for multi-stage bladed disks, and extend it to stage-wise characterization within the integrated disk–blade–shaft representation. For a blisk stage with *N*_s_ blades, the circumferential variation of a harmonic blade mode can be expressed in a complex form as

(14) $$u_{j}^{\left(s\right)}=ℜ\left\{A^{\left(s\right)}e^{i\left(\frac{2\pi l_{s}}{N_{s}}j+\psi^{\left(s\right)}\right)}\right\},j=0,1,\ldots ,N_{s}\mathrm{-1},$$

where $l_{s}$ is the nodal-diameter (ND) index and $\psi^{\left(s\right)}$ is the phase shift of stages. For the *r*-th global mode of the integrated rotor, the DOFs at corresponding blade locations in each stage are collected into a stage-wise blade characteristic vector.

(15) $$b_{r}^{\left(s\right)}={\left[u_{r,1}^{\left(s\right)},u_{r,2}^{\left(s\right)},\ldots ,u_{r,N_{s}}^{\left(s\right)}\right]}^{T}$$

Which serves as a compact “sub-pattern” describing how the blades of stages participate in the global mode. The corresponding ND-spectrum is obtained via a discrete Fourier transform (DFT)

(16) $$B_{r}^{\left(s\right)}=F_{N_{s}}\left(b_{r}^{\left(s\right)}\right),\;\Gamma_{r}^{\left(s\right)}(q)=\left|B_{r}^{\left(s\right)}[q]\right|,q=0,\ldots ,\frac{N_{s}}{2},$$

And the BCNDS map of stage s is constructed by stacking $\Gamma_{r}^{\left(s\right)}$ over the modal order *r*:

(17) $$S^{\left(s\right)}(r,q)=\Gamma_{r}^{\left(s\right)}(q),\;r=1,\ldots ,R.$$

A useful scalar descriptor for inter-stage coupling is the ND-energy concentration ratio

(18) $$\eta_{r}^{\left(s\right)}=\frac{max_{q}S^{\left(s\right)}{\left(r,q\right)}^{2}}{\sum S^{\left(s\right)}{\left(r,q\right)}^{2}},$$

where lower $\eta_{r}^{\left(s\right)}$ generally indicates broader ND participation and stronger coupling/mixing across circumferential harmonics.

**Figure 7:**
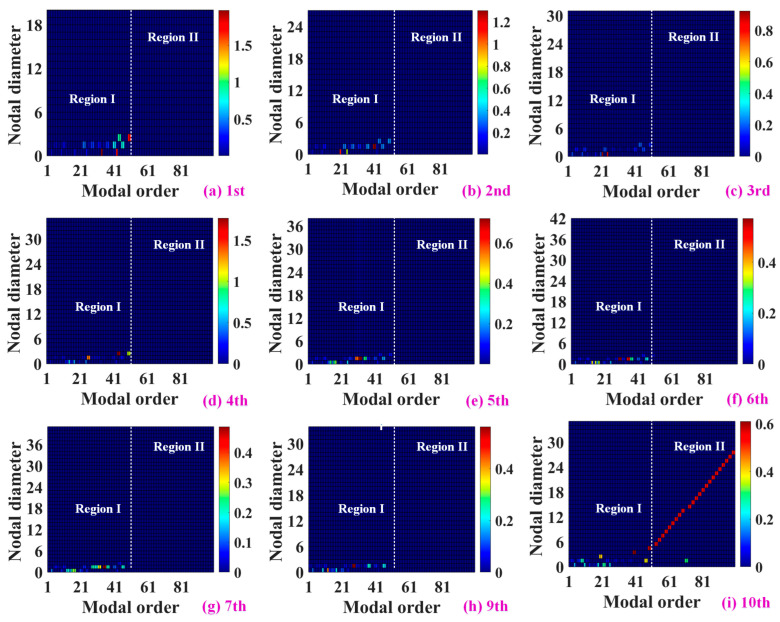
Blade characteristic nodal-diameter spectrum (BCNDS) of the integrated blisk–shaft rotor for Stages 1–10.

Figure 7 presents the BCNDS maps of Stages 1–10, where the modal spectrum is consistently divided into Region I (low-order modes) and Region II (higher-order modes). In Region I, corresponding to low modal orders, the BCNDS maps of all stages exhibit clear energy concentration within a narrow range of low nodal diameters, predominantly ND = 0–2. This behavior indicates that the vibration response in this region is governed by multi-stage disk–shaft coupled motion, in which blades participate collectively through low-order circumferential harmonics imposed by global shaft bending and axial deformation. The dominance of low nodal diameters confirms that strong inter-stage coupling is primarily a low-order phenomenon, consistent with the repeated and closely spaced frequencies observed in the prestressed modal spectrum.

As the modal order increases into Region II, the BCNDS characteristics change markedly. For most stages, the nodal-diameter energy becomes highly dispersed with no pronounced dominant components, indicating a progressive loss of coherent disk–shaft–blade coupling and a transition toward locally confined blade or disk-dominated vibration. Notably, an exception is observed in the tenth-stage blisk (turbine stage), where a distinct ridge-like distribution spanning nodal diameters approximately from ND = 5 to 28 emerges.

Taken together, the BCNDS results establish a clear physical picture: strong coupling in integrated blisk–shaft rotors is concentrated in low-order, low-nodal-diameter modes, where repeated frequencies and prestress-induced stiffness redistribution jointly amplify global interaction across stages. As the nodal diameter increases, the circumferential coherence of blade motion diminishes, and the overall coupling strength decreases. This stage-resolved spectral evidence explains why single-blisk or single-stage analyses are inherently insufficient, as they fail to capture the low-ND, multi-stage coupled dynamics that dominate the most critical vibration regimes of aero-engine rotors under operating conditions.

## 3. Stochastic Modeling and Uncertainty Analysis of Integrated Blisk–Shaft Rotor Dynamics

The deterministic modal analysis presented in Section 2.4 reveals that the vibration behavior of the integrated blisk–shaft rotor is dominated by low-order, low-nodal-diameter coupled modes, which are further complicated by repeated frequencies and pronounced prestress effects. These features indicate that the dynamic response of the system is highly sensitive to parametric uncertainties. Motivated by these observations, this section focuses on the stochastic modeling and uncertainty analysis of the integrated blisk–shaft rotor, with particular emphasis on the most dynamically critical modal families.

### 3.1. Stochastic Modeling and Definition of Uncertain Parameters

Motivated by the modal analysis presented in Section 2.4, it becomes evident that the vibration behavior of the integrated blisk–shaft rotor is governed by strong multi-stage coupling, repeated modes, and prestress-induced modal interactions. These characteristics indicate a pronounced sensitivity of the global dynamic to small variations in structural and material properties, thereby necessitating a rigorous stochastic modeling framework.

In practical aero-engine applications, uncertainties arise primarily from material property dispersion, manufacturing tolerances, and operational variability. For integrated blisk–shaft rotors, such uncertainties are not confined to isolated components but propagate across multiple stages through the continuous shaft and disk structure. In this study, the stochastic modeling focuses on material-related uncertainties, which are known to play a dominant role in modulating modal characteristics under high rotational speeds.

Specifically, the following parameters are considered as random variables: Young’s modulus *E*, reflecting variability in material stiffness; material density *ρ*, governing mass distribution and inertia effects; Poisson’s ratio ν, influencing coupled deformation behavior; and the rotational speed **Ω**, which directly controls centrifugal stiffening and rotational softening effects and plays a critical role in shaping the prestressed modal characteristics of the integrated blisk–shaft rotor. These parameters are modeled as statistically independent random variables with prescribed probability distributions, calibrated to represent realistic manufacturing and material dispersion levels. The stochastic parameter vector is defined as

(19) $$\xi ={\left[E_{1},\rho_{1},\nu_{1},E_{2},\rho_{2},\nu_{2},E_{3},\rho_{3},\nu_{3},\Omega \right]}^{T}$$

### 3.2. Surrogate Modeling for Stochastic Modal Prediction

As discussed in Section 3.1, the stochastic response of the integrated blisk–shaft rotor is primarily governed by low-order, low-nodal-diameter coupled modes that exhibit strong sensitivity to material and structural uncertainties. Direct Monte Carlo evaluation of these modal characteristics using high-fidelity finite element or reduced-order models remains computationally prohibitive, particularly when multiple uncertain parameters are considered. To enable efficient uncertainty propagation and sensitivity analysis, surrogate modeling techniques are introduced to approximate the implicit mapping between uncertain input parameters and modal responses.

In this study, two representative surrogate models are adopted and compared: the Kriging (Gaussian process) model and the artificial neural network (ANN) model. These two approaches differ fundamentally in their theoretical foundations and approximation mechanisms, providing complementary capabilities for stochastic modal analysis.

It should be emphasized that both surrogate models are constructed using the same training dataset and are applied to the same set of target modal quantities. The objective is not to assign different frequency ranges to different surrogate models, but rather to systematically compare their prediction accuracy, robustness, and suitability for stochastic vibration analysis of integrated rotor systems.

#### 3.2.1. Kriging-Based Surrogate Model

Kriging is a probabilistic surrogate modeling technique that combines a global regression trend with a localized stochastic deviation. For a given set of uncertain input parameters

(20) $$\xi ={\left[\xi_{1},\xi_{2},\ldots ,\xi_{d}\right]}^{T}$$

The Kriging approximation of a modal quantity of interest (e.g., natural frequency or modal participation factor) is expressed as

(21) $$\hat{y}(\xi )=f^{T}(\xi )\beta +Z(\xi )$$

where $f^{T}(\xi )$ denotes a vector of predefined basis functions, β is the corresponding regression coefficient vector, and Z(ξ) represents a zero-mean Gaussian random process with covariance

(22) $$\mathrm{Cov}\left[Z\left(\xi_{i}\right),Z\left(\xi_{j}\right)\right]=\sigma^{2}R\left(\xi_{i},\xi_{j}\right)$$

The correlation function *R*(**.**) characterizes the spatial correlation between sampling points in the stochastic space. A commonly adopted Gaussian correlation function is given by

(23) $$R\left(\xi_{i},\xi_{j}\right)=\exp\left(-\sum_{k=1}^{d}\theta_{k}{\left|\xi_{i,k}-\xi_{j,k}\right|}^{2}\right)$$

where $\theta_{k}$ are hyperparameters controlling the smoothness of the surrogate model along each uncertain dimension. These hyperparameters are typically identified by maximizing the likelihood function based on training samples generated from the reduced-order model.

Owing to its interpolation property and built-in uncertainty quantification capability, Kriging provides high accuracy for smooth response surfaces and serves as a reliable benchmark surrogate for stochastic modal prediction.

#### 3.2.2. Artificial Neural Network Surrogate Model

**Figure 8:**
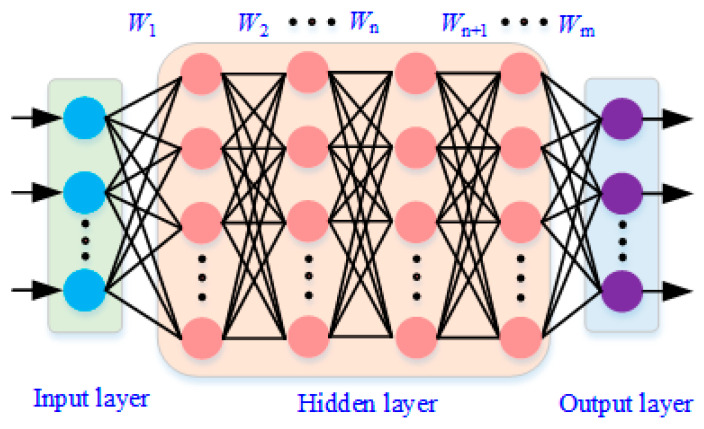
Architecture of the artificial neural network (ANN) surrogate model.

Artificial neural networks offer a flexible, data-driven alternative capable of capturing highly nonlinear relationships between input uncertainties and modal responses. In this study, a fully connected feedforward neural network is adopted, as illustrated in Figure 8, consisting of an input layer, multiple hidden layers, and an output layer.

Let $x\in {\mathbb{R}}^{d}$ denote the vector of uncertain input parameters. The forward propagation of the ANN can be written as

(24) $$h^{\left(l\right)}=\phi \left(W^{\left(l\right)}h^{\left(\mathrm{l-}1\right)}+b^{\left(l\right)}\right),\;l=1,2,\ldots ,L$$

where $h^{\left(0\right)}=x$, $W^{\left(l\right)}$ and $b^{\left(l\right)}$ are the weight matrix and bias vector of the *l*-th layer, respectively, and ϕ (**.**) denotes a nonlinear activation function. The network output is given by

(25) $$\hat{y}(x)=W^{\left(L+1\right)}h^{\left(L\right)}+b^{\left(L+1\right)}$$

The ANN parameters are optimized by minimizing the mean squared error between the predicted and reference modal quantities

(26) $$L=\frac{1}{N}\sum_{i=1}^{N}{\left(y_{i}-\hat{y}\left(x_{i}\right)\right)}^{2}$$

using gradient-based backpropagation algorithms.

Compared with Kriging, ANNs do not rely on predefined correlation functions and are particularly effective in handling high-dimensional input spaces and strong nonlinearities. However, their prediction accuracy is more sensitive to training data quality and network architecture.

### 3.3. Uncertainty Analysis Framework Based on Reduced-Order Modeling and Surrogate Learning

**Figure 9:**
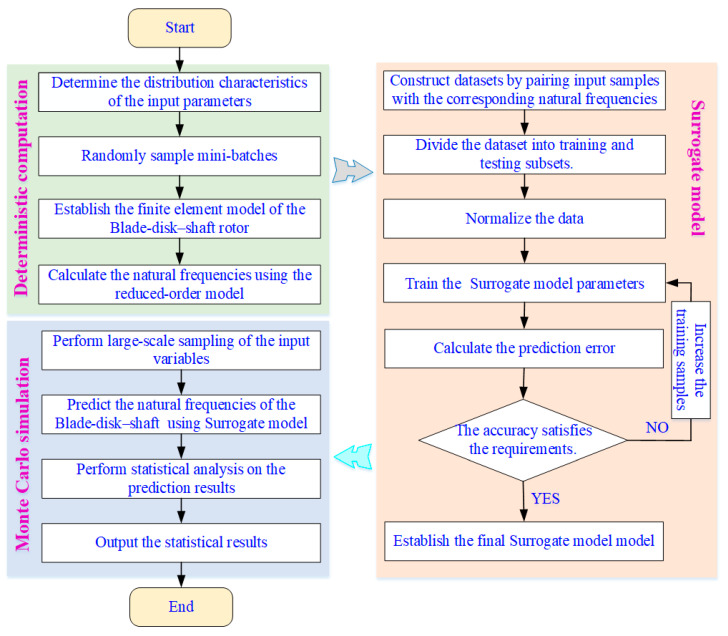
Framework of the uncertainty analysis for the integrated blisk–shaft rotor based on reduced-order modeling and surrogate models.

To efficiently quantify the vibration uncertainty of the integrated blisk–shaft rotor under parametric randomness, a hybrid uncertainty analysis framework is established by coupling reduced-order modeling (ROM) with data-driven surrogate models, as shown in Figure 9. The core idea is to exploit the high-fidelity yet low-cost ROM to generate accurate training data, and subsequently employ surrogate learning to enable large-scale uncertainty propagation that would be computationally infeasible using full finite element simulations.

In this framework, a limited number of input parameter samples are first generated according to the prescribed probability distributions, and the corresponding natural frequencies are computed using the prestress-consistent reduced-order model developed in Section 2.3. This step ensures that the essential dynamic characteristics of the integrated rotor, including multi-stage coupling and prestress effects, are faithfully retained in the training dataset. The sampled input–output pairs are then used to construct surrogate models that approximate the nonlinear mapping between uncertain parameters and modal frequencies.

Once the surrogate model is established and validated, it is employed to perform large-scale Monte Carlo sampling, enabling efficient propagation of input uncertainties to the modal responses. Statistical quantities of interest, such as probability distributions, moments, and dispersion characteristics of the natural frequencies, are subsequently extracted to characterize the vibration uncertainty of the system.

By combining ROM-based data generation with surrogate-based uncertainty propagation, the proposed framework achieves a favorable balance between accuracy and computational efficiency. More importantly, it provides a scalable tool for uncertainty quantification of dynamically critical low-order, low-nodal-diameter modes identified in Section 2.4, forming a solid basis for stochastic analysis and reliability-oriented rotor design.

## 4. Uncertainty Analysis Results

### 4.1. Definition of Random Input Variables

To investigate the stochastic vibration characteristics of the integrated blisk–shaft rotor, key material and operational parameters are treated as random variables. In this study, the Young’s modulus of blades at different stages, material density, Poisson’s ratio, and the rotational speed are selected as uncertainty sources, as summarized in Table 5. All random variables are modeled as Gaussian distributions characterized by their nominal mean values and coefficients of variation (CV).

Table 5 lists the adopted uncertainty description for each parameter. It should be emphasized that, in the absence of component- and batch-specific joint statistical information (e.g., covariance matrices or measured correlation coefficients for the investigated alloys and manufacturing routes), an independence (uncorrelated) assumption is employed here as a baseline uncertainty model to ensure a transparent and reproducible uncertainty quantification setting. Such baseline probabilistic modeling and uncertainty propagation strategies have been widely adopted in vibration analyses of mistuned bladed disks/blisks and related aero-engine structures when detailed joint statistics are unavailable [6,39,40]. We acknowledge that material properties within the same component (e.g., potential correlations between *E* and *ρ*) may exist in practice and may influence uncertainty propagation; incorporating correlated uncertainties (e.g., via covariance-matrix-based multivariate sampling or copula-based approaches) is straightforward within the present sampling-based framework and will be considered in future work when relevant statistical data become available. Such correlations may increase or decrease the predicted frequency variance depending on the sign and strength of correlation, and may also affect the relative importance ranking of input variables. In particular, coupled stiffness–mass correlations can modify the effective dispersion of modal frequencies compared with the uncorrelated baseline.

With this baseline uncertainty specification, the output quantities of interest are chosen as the first ten natural frequencies of the integrated blisk–shaft rotor. These modes have been identified in Section 2 as the most dynamically critical ones due to their low-order characteristics, low nodal diameters, and pronounced disk–blade–shaft coupling. The resulting stochastic frequency samples are then used for subsequent uncertainty propagation, surrogate modeling, and sensitivity analyses.

### 4.2. Surrogate Model Accuracy Under Small-Sample Conditions

To evaluate the predictive capability of different surrogate models under limited training data, a small-batch sampling strategy with 100 random samples is adopted. For each sample, the reduced-order finite element model is employed to compute the corresponding natural frequencies, which serve as reference responses for surrogate model construction

**Figure 10:**
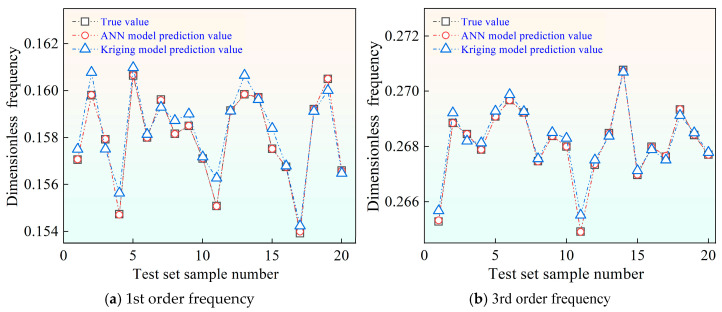

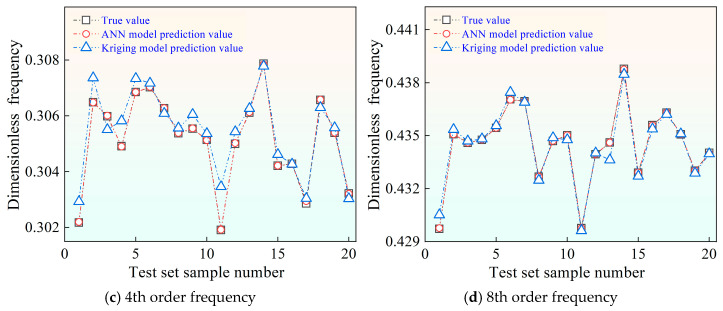
Comparison of natural frequency predictions by ANN and Kriging surrogate models.

Two surrogate models—Kriging and artificial neural networks (ANNs)—are trained using the same dataset and then validated against an independent testing set. The detailed ANN configuration and training settings are summarized in Table 6. The Kriging model follows standard ordinary Kriging with a Gaussian correlation. Using the same fixed settings, Kriging and ANN are trained on an identical 100-sample dataset under the same split to ensure a fair comparison. Figure 10a–d present representative comparisons between true values and surrogate predictions for selected low-order natural frequencies.

While both surrogate models successfully capture the overall trends of frequency variation induced by parameter uncertainty, noticeable differences in predictive accuracy are observed. The Kriging model exhibits localized deviations, particularly near extrema and in regions where the frequency response shows stronger nonlinearity. In contrast, the ANN predictions remain consistently closer to the reference values across the entire testing set, indicating improved generalization capability under small-sample conditions.

To further quantify the prediction accuracy, the mean absolute percentage error (MAPE) is introduced as a scalar evaluation metric. For the *k*-th natural frequency, the MAPE is defined as

(27) $${\mathrm{MAPE}}_{k}=\frac{1}{N_{\mathrm{test}\;}}\sum_{i=1}^{N_{\mathrm{test}\;}}\left|\frac{f_{k,i}^{\mathrm{pred}}-f_{k,i}^{\mathrm{ref}}}{f_{k,i}^{\mathrm{ref}}}\right|\times 100\%$$

where $f_{k,i}^{\mathrm{pred}}$ and $f_{k,i}^{\mathrm{ref}}$ denote the surrogate-predicted and reference natural frequencies for the *i*-th testing sample, respectively, and $N_{\mathrm{test}\;}$ is the number of testing samples.

Table 7 summarizes the MAPE values of the first ten natural frequencies predicted by the two surrogate models. It is observed that the ANN consistently achieves lower MAPE values than the Kriging model across all considered modes. The improvement is particularly pronounced for low-order, strongly coupled modes, which are characterized by low nodal diameters and enhanced disk–shaft–blade interaction.

These results demonstrate that, under small-sample conditions, the ANN surrogate provides superior accuracy and robustness in approximating the nonlinear mapping between uncertain input parameters and modal characteristics. Consequently, the ANN-based surrogate model is adopted in the subsequent large-scale Monte Carlo simulations for stochastic vibration analysis of the integrated blisk–shaft rotor.

### 4.3. Statistical Characterization of Stochastic Natural Frequencies

To further elucidate the statistical characteristics of the stochastic natural frequencies of the integrated blisk–shaft rotor, a large-scale Monte Carlo simulation is conducted based on the reduced-order model and the ANN surrogate established in Section 3.2 and Section 3.3. For each realization of the random input variables, the first several natural frequencies are evaluated, yielding an ensemble of stochastic frequency samples for each modal order.

#### 4.3.1. Probability Distribution Modeling of Natural Frequencies

**Figure 11:**
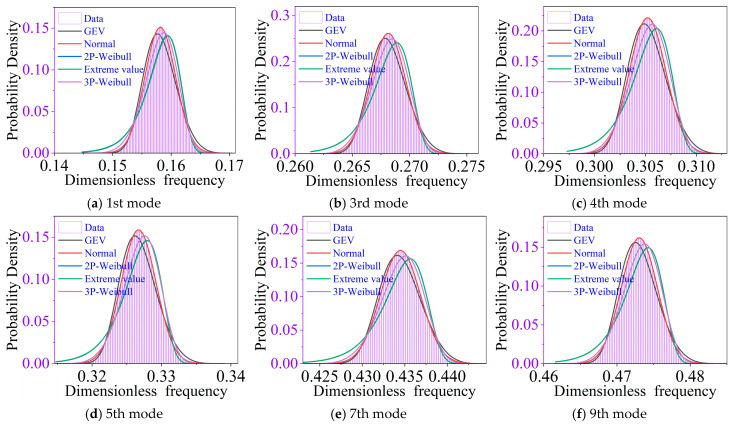
Probability density function of natural frequency.

Figure 11 presents the probability density histograms of representative low-order natural frequencies together with fitted probability distribution functions. To assess the most appropriate statistical model, several commonly used distributions are considered, including the normal distribution, two-parameter Weibull distribution, three-parameter Weibull distribution, extreme value distribution, and generalized extreme value (GEV) distribution.

Taking representative probability density functions as references, the statistical distribution of the random natural frequencies is examined using the normal distribution and the three-parameter Weibull distribution.

For the normal distribution, the probability density function (PDF) of a random natural frequency *X* is given by

(28) $$f_{X}(x)=\frac{1}{\sigma \sqrt{2\pi }}\exp\left[-\frac{{\left(x-\mu \right)}^{2}}{2\sigma^{2}}\right]$$

where *μ* and *σ* denote the mean value and standard deviation of the random natural frequency, respectively. The normal distribution serves as a baseline model to assess the symmetry and dispersion characteristics of the frequency variability induced by parametric uncertainties.

To account for potential skewness and tail behavior in the frequency distribution, the three-parameter Weibull distribution is further considered, whose PDF is expressed as

(29) $$f_{X}(x)=\frac{k}{\lambda }{\left(\frac{x-\gamma }{\lambda }\right)}^{k-1}\exp\left[-{\left(\frac{x-\gamma }{\lambda }\right)}^{k}\right],\;x>\gamma ,$$

where *k*, *λ*, and *γ* represent the shape, scale, and location parameters, respectively. Compared with two-parameter distributions, the inclusion of the location parameter enables the three-parameter Weibull model to flexibly capture asymmetric distributions and boundary effects commonly observed in vibration-related random responses.

For each modal order, the unknown parameters of the candidate distributions are estimated using the maximum likelihood estimation (MLE) method. The fitted curves are then compared with the Monte Carlo histograms to evaluate their goodness of fit.

As illustrated in Figure 11, although slight skewness can be observed for certain modes, the normal distribution consistently provides an accurate approximation of the stochastic natural frequency distributions across all considered modal orders. In contrast, extreme value–type distributions and Weibull distributions exhibit noticeable deviations in the tail regions, particularly for modes dominated by global disk–shaft coupling. These results indicate that, unlike mistuned forced-response amplification factors, the stochastic variability of natural frequencies is primarily governed by accumulated linear perturbations, leading to near-Gaussian behavior.

#### 4.3.2. Statistical Moments and Sensitivity to Input Variability

Based on the fitted distributions, the statistical moments of the stochastic natural frequencies are evaluated. Table 8 summarizes the mean values and standard deviations of the first ten natural frequencies under different coefficients of variation of the random input parameters.

The statistics in Table 8 reveal clear and robust trends in the stochastic modal characteristics of the integrated blisk–shaft rotor. For all modes, the mean natural frequencies remain nearly unchanged with increasing input coefficient of variation (CV), indicating that parameter uncertainty primarily affects dispersion rather than shifting the central frequency values.

In contrast, the standard deviation exhibits a systematic increase with modal order, demonstrating that higher-order modes are progressively more sensitive to material and operational uncertainties. This sensitivity amplification is especially evident for modes associated with stronger blade–disk–shaft coupling, where uncertainty propagation is more pronounced.

Moreover, for a given modal order, the growth of the standard deviation with respect to the input CV is monotonic but not strictly linear. The incremental increase in dispersion slightly diminishes at higher CV levels, suggesting a saturation effect governed by the combined influence of modal coupling and structural stiffness redistribution. These observations confirm that the stochastic behavior of the integrated rotor is dominated by variance amplification rather than mean bias, and that uncertainty sensitivity depends jointly on modal order and coupling intensity.

### 4.4. Sensitivity Analysis of Stochastic Natural Frequencies

Following the statistical characterization of stochastic natural frequencies presented in Section 4.3, a sensitivity analysis is conducted to further quantify the relative influence of uncertain input parameters on the modal variability of the integrated blisk–shaft rotor. Since the exact functional relationship between the input uncertainties and modal responses is generally unknown and potentially nonlinear, a nonparametric rank-based sensitivity measure is adopted.

The sensitivity of the stochastic natural frequencies to each uncertain input parameter is evaluated using the Spearman rank correlation coefficient, which measures the monotonic dependence between input and output variables without assuming linearity. For the *j*-th input variable and a given modal frequency response, the Spearman correlation coefficient is defined as

(30) $$r_{j}=\frac{\sum_{i=1}^{n}\left(R_{ij}-{\bar{R}}_{j}\right)\left(Q_{i}-\bar{Q}\right)}{\sqrt{\sum_{i=1}^{n}{\left(R_{ij}-{\bar{R}}_{j}\right)}^{2}}\sqrt{\sum_{i=1}^{n}{\left(Q_{i}-\bar{Q}\right)}^{2}}}$$

where $R_{ij}$ denotes the rank of the *i*-th sample of the *j*-th input variable, $Q_{i}$ is the rank of the corresponding output natural frequency, and *n* is the total number of samples.

Under practical Monte Carlo sampling, Equation (30) can be simplified as

(31) $$r_{j}=1-\frac{6\sum_{i=1}^{n}{\left(R_{ij}-Q_{i}\right)}^{2}}{n\left(n^{2}-1\right)}$$

**Figure 12:**
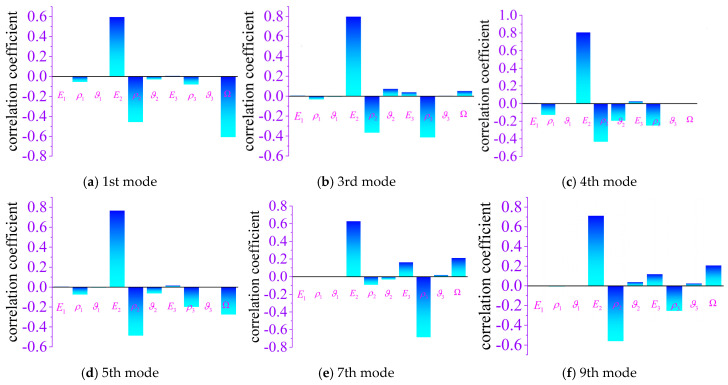
Sensitivity of natural frequency.

Figure 12 illustrates the sensitivity indices of selected natural frequencies with respect to the uncertain parameters listed in Table 5. A positive sensitivity coefficient indicates that the natural frequency increases with an increase in the corresponding parameter, whereas a negative value implies a decreasing trend. The absolute magnitude of the sensitivity coefficient reflects the relative importance of each parameter in governing frequency variability.

Several important observations can be drawn from Figure 12. First, the sensitivity patterns vary markedly with modal order, indicating that different vibration modes are governed by distinct physical mechanisms. For the low-order global modes, particularly the first and fifth modes, the Young’s modulus and density of the compressor disk exhibit the highest absolute sensitivity values. This suggests that the stiffness–mass characteristics of the disk play a dominant role in determining the natural frequencies of global bending-dominated modes. In contrast, the elastic modulus and density of the compressor blades contribute only marginally to these low-order modes, reflecting the fact that blade deformation is secondary in globally coupled shaft–disk vibrations.

Second, the influence of rotational speed, which represents centrifugal prestress effects, is strongly mode-dependent. For the global first bending mode and bending–torsion coupled modes, the sensitivity to rotational speed is pronounced and negative, indicating that the introduction of centrifugal prestress leads to a reduction in the corresponding natural frequencies. This behavior highlights that prestress does not universally stiffen the structure; instead, it may induce geometric softening for certain global bending-dominated vibration forms. Conversely, for axial and torsional modes, the sensitivity to rotational speed is relatively small, implying that these vibration forms are less affected by prestress-induced stiffness redistribution.

Third, the Poisson’s ratios of both blades and disks exhibit consistently low sensitivity magnitudes across most modal orders. Compared with Young’s modulus and density, their contribution to natural frequency variability is negligible within the considered uncertainty range. This indicates that Poisson’s ratio plays a secondary role in governing stochastic frequency dispersion and may be treated as a lower-priority random variable in uncertainty modeling for the present system.

Overall, the sensitivity analysis confirms that the stochastic behavior of the integrated blisk–shaft rotor is primarily driven by variations in stiffness and mass parameters of the disk components, together with mode-dependent centrifugal prestress effects. The strong dependence of global bending and bending–torsion coupled modes on rotational speed further underscores the necessity of incorporating prestress-consistent modeling in stochastic vibration analysis. These results provide a clear physical basis for parameter prioritization in uncertainty quantification and reliability assessment of aero-engine rotors.

To further strengthen the sensitivity conclusions, we additionally performed a variance-based global sensitivity analysis using Sobol indices for the first natural frequency *f*_1_. Spearman’s rank correlation is useful for screening monotonic relationships, but it does not quantify variance contributions or interaction effects. Therefore, we report the first-order Sobol index *S_i_* and the total-effect index *S_Ti_*, which measure the main contribution of each uncertain input and its overall contribution, including interactions, respectively. To keep the additional analysis concise while ensuring robustness, Sobol indices were evaluated using the trained ANN surrogate, enabling efficient sampling without repeated FE/ROM runs. The results are summarized in Table 9. The difference *S_Ti_ − S_i_* reflects the strength of interaction effects involving the *i*-th parameter.

For the representative frequency *f*_1_ (the global first bending mode), the variance-based Sobol indices computed using the ANN surrogate indicate a highly concentrated uncertainty contribution. The dominant factors are the compressor-disk Young’s modulus *E_2_* and the rotational speed Ω, with first-order indices *S_E_*_2_ = 0.372 and *S*_Ω_ = 0.377, respectively, followed by the compressor-disk density *ρ*_2_ (S*_ρ_*_2_ = 0.223). Collectively, these three variables explain approximately 97.2% of the output variance of *f*_1_ through first-order effects, demonstrating that the first bending frequency is primarily governed by the compressor-disk stiffness–mass properties and the operating-speed-induced prestress effect. Moreover, the total-effect indices are only slightly larger than the corresponding first-order indices (e.g., *S_T,E_*_2_ = 0.378, *S_T,Ω_* = 0.386, S_T,*ρ*2_ = 0.227), and the interaction-related contributions *S_T,i_−S_i_* remain small for all parameters (maximum ≈ 0.009 for Ω). This suggests that uncertainty propagation in *f*_1_ is dominated by nearly additive main effects, while higher-order interactions play a secondary role under the considered uncertainty level.

To provide an intuitive interpretation of directionality, Figure 12a reports the Spearman rank correlation coefficients between the uncertain inputs and *f*_1_. The results show that *E_2_* exhibits the strongest positive monotonic influence on *f*_1_, whereas *ρ*_2_ and Ω display pronounced negative influences; the remaining parameters are only weakly correlated with *f*_1_. This qualitative ranking and sign agree well with the Sobol-based importance measures and support a clear physical explanation: increasing *E*_2_ raises the effective stiffness and thus increases the bending frequency, while increasing *ρ*_2_ reduces the frequency through the mass effect, and variations in Ω significantly affect *f*_1_ through speed-dependent prestress (stress-stiffening) mechanisms.

Taken together, Sobol indices provide a rigorous variance decomposition to quantify parameter importance and confirm the limited role of interactions for *f*_1_, whereas Spearman coefficients offer a complementary trend-based validation and sign interpretation. These findings are also consistent with the modal nature of *f*_1_ as a global first bending mode dominated by shaft–disk backbone deformation rather than blade-local deformation, explaining why blade- and turbine-stage material parameters (*E*_1_, *ρ*_1_, *ν*_1_, *E*_3_, *ρ*_3_, *ν*_3_) contribute negligibly (*S_i_* < 0.01 individually).

## 5. Conclusions

This study establishes an efficient and physically grounded framework for stochastic vibration analysis of integrated blisk–shaft rotors by coupling prestress-consistent reduced-order modeling with data-driven surrogate techniques. A multi-level prestressed substructuring reduced-order model (MPS-ROM) is developed to retain essential disk–blade–shaft coupling while achieving substantial computational reduction, and its accuracy is rigorously confirmed through frequency error and MAC assessments.

Deterministic modal analysis demonstrates that the coupled dynamic behavior of integrated blisk–shaft rotors is governed by low-order, low-nodal-diameter modes, accompanied by closely spaced and repeated frequencies induced by cyclic symmetry and axial continuity. Centrifugal prestress is shown to play a dual role, not only shifting modal frequencies but also reshaping modal coupling.

To enable efficient uncertainty propagation, artificial neural network (ANN) surrogates trained on small-sample ROM data are employed and systematically compared with Kriging models. The ANN surrogates consistently deliver higher prediction accuracy for strongly coupled low-order modes, particularly under pronounced prestress effects and nonlinear parameter interactions.

Monte Carlo-based uncertainty analysis reveals that the stochastic distributions of natural frequencies are well approximated by normal distributions, with dispersion levels increasing with modal order and coupling intensity, while mean values remain close to deterministic predictions. This confirms that modal uncertainty in integrated rotors is primarily governed by variance amplification rather than mean drift.

Sensitivity analysis further identifies clear parameter-dependent mechanisms. The elastic modulus and density of compressor disks dominate the uncertainty of low-order global modes, whereas blade material properties contribute secondarily. Prestress induced by rotational speed exhibits mode-dependent influence, enhancing or reducing frequencies depending on vibration form, while Poisson’s ratio plays a negligible role within the examined uncertainty range.

Overall, the proposed framework provides a robust and scalable tool for stochastic dynamic analysis of advanced aero-engine rotors. The results offer clear guidance for parameter prioritization, uncertainty-aware design, and reliability assessment of integrated blisk–shaft systems operating under realistic uncertain conditions.

## Figures and Tables

**Table 1:** Number of blades of compressor and turbine.

Stage Number	1	2	3	4	5	6	7	8	9	10
Number of blades	38	53	60	68	75	82	82	80	76	68

**Table 2:** Material properties of the blisk-shaft integration rotor.

	Compressor Blade	Compressor Disk	Turbine Blade and Disk
Density (kg/m^3^)	4400	4600	8200
Young modulus (Pa)	1.14 × 10^11^	1.15 × 10^11^	1.66 × 10^11^
Poisson ratio	0.3	0.3	0.3

**Table 3:** Extended ROM validation under additional rotational speeds and material perturbations.

Case	Ω (rad/s)	Material Perturbation	Modes	Max Freq. Error (%)	Min MAC
1	0	nominal	1–50	2.55	0.95
2	1188.3	nominal	1–50	2.74	0.92
3	500	nominal	1–50	2.63	0.93
4	1188.3	Turbine disk *E* + 2%	1–50	2.71	0.92
5	1188.3	Compressor blade *ρ* + 2%	1–50	2.68	0.92

**Table 4:** Modal classification and dimensionless natural frequencies of the integrated blisk–shaft rotor under different rotational speeds.

0 rad/s			1188.3 rad/s		
Mode Order	Dimensionless Frequency	Mode Shape	Mode Order	Dimensionless Frequency	Mode Shape
1–2	0.21471	Global shaft–disk coupled bending mode (1st)	1–2	0.15817	Global shaft–disk coupled bending mode (1st)
3	0.26304	Global shaft–disk axial translational mode	3	0.26814	Global shaft–disk axial translational mode
4	0.30531	Global shaft–disk coupled torsional mode (1st)	4	0.30521	Global shaft–disk coupled torsional mode (1st)
5–6	0.35569	Global bending–torsional coupled mode	5–6	0.32670	Global bending–torsional coupled mode
7–8	0.41169	Turbine blisk-dominated radial bending mode	7–8	0.43448	Turbine bliisk-dominated radial bending mode
9	0.44096	Global shaft–disk coupled torsional mode (2nd)	9	0.47304	Global shaft–disk coupled torsional mode (2nd)
10–11	0.51262	Global shaft–disk coupled bending mode (2nd)	10–11	0.49588	Global shaft–disk coupled bending mode (2nd)

**Table 5:** Characteristic of random variables of the integrated blisk–shaft rotor.

Types	Random Variables	Mean Values	CV
Material properties	Young modulus of the compressor blade *E*_1_/(Pa)	1.14 × 10^11^	0.01
	Density of the compressor blade *ρ*_1_/(kg/m^3^)	4400	0.01
	$\mathrm{Poisson}\;\mathrm{ratio}\;\mathrm{of}\;\mathrm{the}\;\mathrm{compressor}\;\mathrm{blade}\;\vartheta_{1}$	0.3	0.01
	Young modulus of the Compressor disk *E*_2_/(Pa)	1.15 × 10^11^	0.01
	Density of the compressor disk *ρ*_2_/(kg/m^3^)	4600	0.01
	$\mathrm{Poisson}\;\mathrm{ratio}\;\mathrm{of}\;\mathrm{the}\;\mathrm{compressor}\;\mathrm{disk}\;\vartheta_{2}$	0.3	0.01
	Young modulus of the turbine blade and disk *E*_3_/(Pa)	1.66 × 10^11^	0.01
	Density of the turbine blade and disk *ρ*_3_/(kg/m^3^)	8200	0.01
	$\mathrm{Poisson}\;\mathrm{ratio}\;\mathrm{of}\;\mathrm{the}\;\mathrm{turbine}\;\mathrm{blade}\;\mathrm{and}\;\mathrm{disk}\;\vartheta_{3}$	0.3	0.01
Operating condition	Rotational speed Ω/(rad/s)	1188.3	0.01

Note: In this work, a uniform CV = 0.01 is adopted as a baseline setting for demonstration and for ensuring comparability across parameters, rather than being calibrated from a specific manufacturing dataset. In practical applications, parameter-dependent dispersions informed by manufacturing tolerances or experimental characterization can be incorporated straightforwardly within the same framework once such data are available.

**Table 6:** ANN configuration and training settings.

Item	Setting in This Study	Item	Setting in This Study
Network type	Feedforward neural network	Testing samples	20 (20%)
Inputs/outputs	10 inputs → 10 outputs	Hidden activation	Tansig
Hidden layers	1	Output activation	Purelin
Neurons per hidden layer	15	Loss function	MSE
Training samples	80 (80%)	Input/Output scaling	mapminmax

**Table 7:** The MAPE values of the first ten natural frequencies predicted by kriging model and ANN.

Mode Order	1	2	3	4	5	6	7	8	9	10
ANN	0.009%	0.010%	0.003%	0.005%	0.004%	0.003%	0.003%	0.003%	0.003%	0.004%
Kriging	0.276%	0.271%	0.072%	0.134%	0.075%	0.071%	0.044%	0.053%	0.064%	0.032%

**Table 8:** Statistics of stochastic natural frequencies under different input coefficient of variation.

	CV = 0.005		CV = 0.01		CV = 0.015		CV = 0.02	
Mode Order	Mean Value	Standard Deviation	Mean Value	Standard Deviation	Mean Value	Standard Deviation	Mean Value	Standard Deviation
1–2	0.15809	0.00227	0.15811	0.00258	0.15813	0.00264	0.15817	0.00270
3	0.26811	0.00139	0.26813	0.00152	0.26814	0.00152	0.26815	0.00155
4	0.30513	0.00178	0.30522	0.00175	0.30521	0.00181	0.30519	0.00184
5–6	0.32666	0.00237	0.32669	0.00250	0.32669	0.00253	0.32668	0.00258
7–8	0.43444	0.00219	0.43445	0.00238	0.43449	0.00234	0.43451	0.00241
9	0.47299	0.00230	0.47306	0.00246	0.47306	0.00246	0.47306	0.00245
10–11	0.495856	0.00297	0.49593	0.00304	0.49588	0.00313	0.49593	0.00315

**Table 9:** Sobol first-order (*S_i_*) and total-effect (*S_Ti_*) indices for the first natural frequency *f*_1_.

Parameter	*S_i_*	*S_Ti_*	(*S_Ti_ − S_i_*)
Young modulus of the compressor blade *E*_1_/(Pa)	0.001276613	0.002499725	0.001223112
Density of the compressor blade *ρ*_1_/(kg/m^3^)	0.004408741	0.005600666	0.001191925
$\mathrm{Poisson}\;\mathrm{ratio}\;\mathrm{of}\;\mathrm{the}\;\mathrm{compressor}\;\mathrm{blade}\;\vartheta_{1}$	0.001618466	0.00162835	9.88442 × 10^−6^
Young modulus of the Compressor disk *E*_2_/(Pa)	0.371912968	0.37839465	0.006481683
Density of the compressor disk *ρ*_2_/(kg/m^3^)	0.222546929	0.226847554	0.004300625
$\mathrm{Poisson}\;\mathrm{ratio}\;\mathrm{of}\;\mathrm{the}\;\mathrm{compressor}\;\mathrm{disk}\;\vartheta_{2}$	0.001995291	0.002149629	0.000154338
Young modulus of the turbine blade and disk *E*_3_/(Pa)	0.001337467	0.003132419	0.001794952
Density of the turbine blade and disk *ρ*_3_/(kg/m^3^)	0.004606564	0.005889363	0.0012828
$\mathrm{Poisson}\;\mathrm{ratio}\;\mathrm{of}\;\mathrm{the}\;\mathrm{turbine}\;\mathrm{blade}\;\mathrm{and}\;\mathrm{disk}\;\vartheta_{3}$	0.002205411	0.003085932	0.00088052
Rotational speed Ω/(rad/s)	0.377340396	0.386371026	0.009030629

## Data Availability

The original contributions presented in this study are included in the article. Further inquiries can be directed to the corresponding authors.
